# Supplementary figures and images for: GPR18 undergoes a high degree of constitutive trafficking but is unresponsive to N-Arachidonoyl Glycine
Source: PeerJ. 2016 Mar 21;4:e1835. doi: 10.7717/peerj.1835 (PMC4806671; doi:10.7717/peerj.1835)

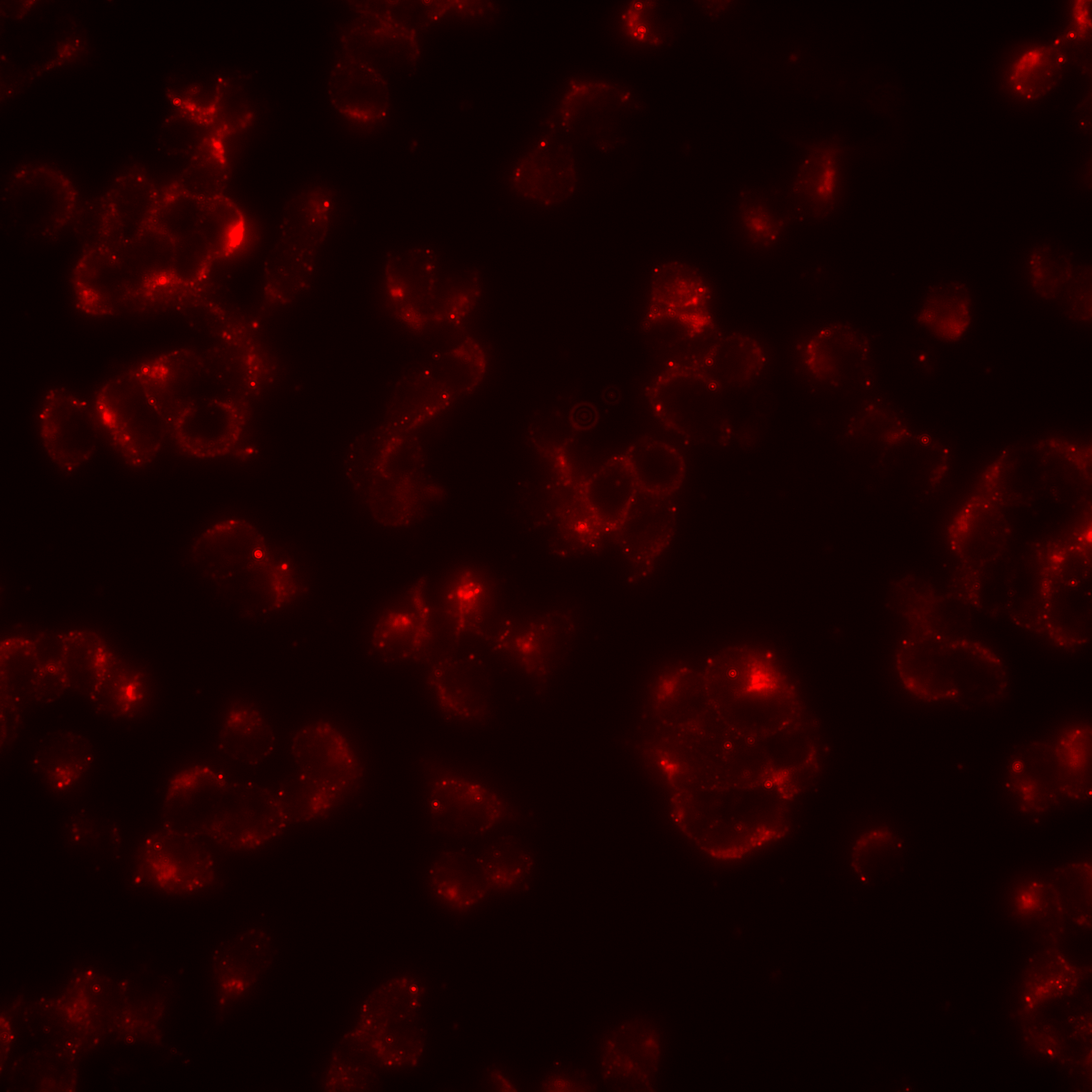

Supplement: Supplemental Information 1 [file peerj-04-1835-s001.tif]

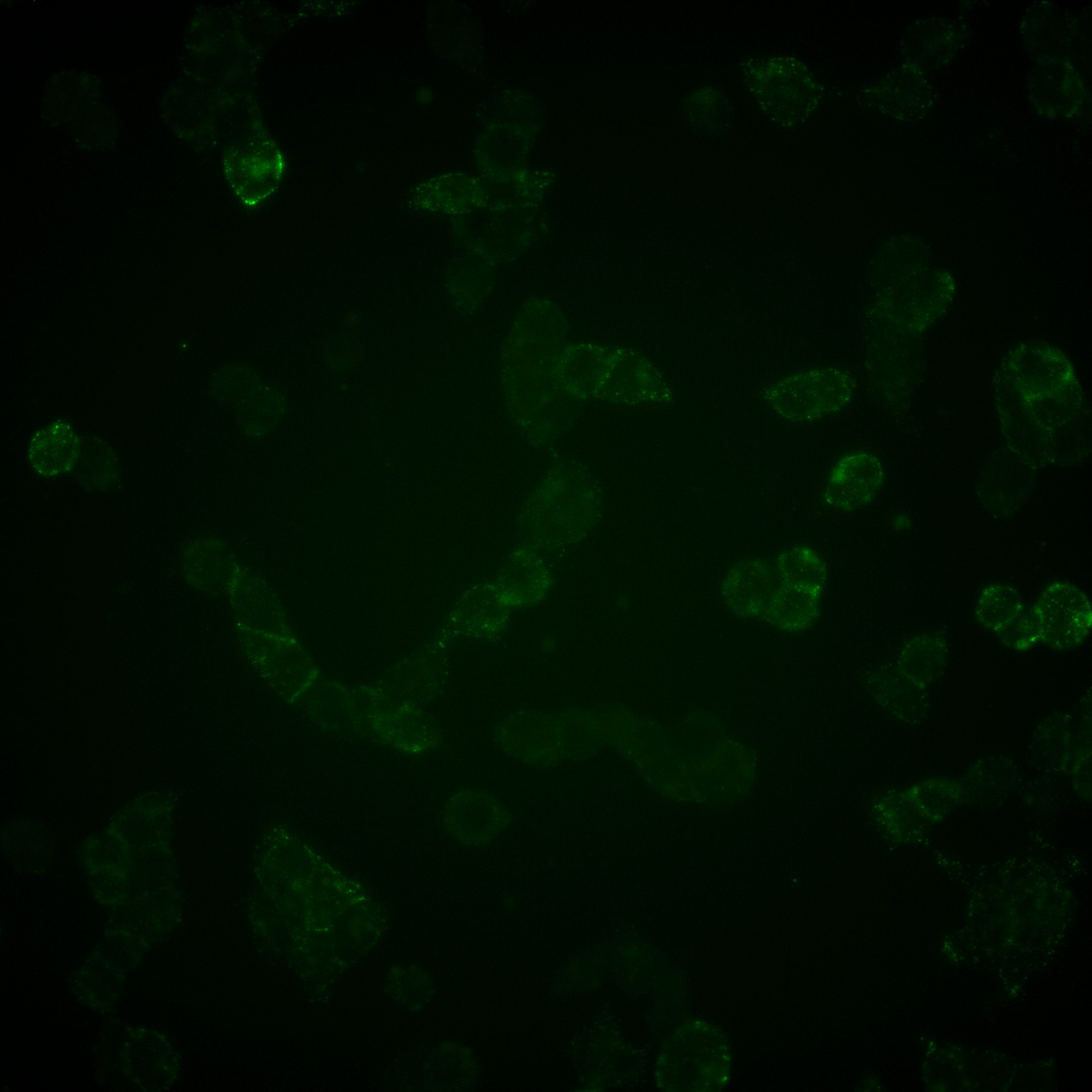

Supplement: Supplemental Information 2 [file peerj-04-1835-s002.tif]

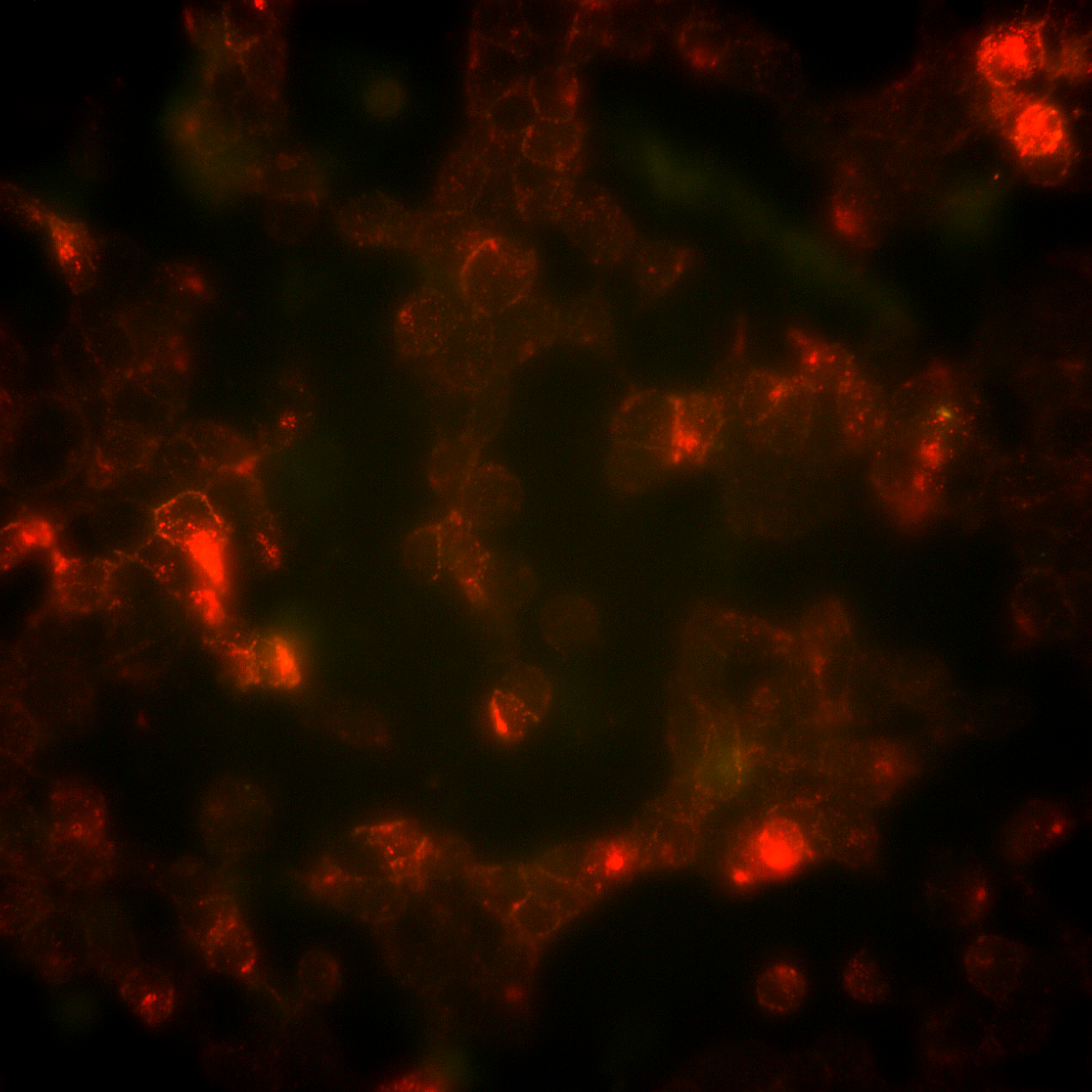

Supplement: Supplemental Information 3 [file peerj-04-1835-s003.tif]

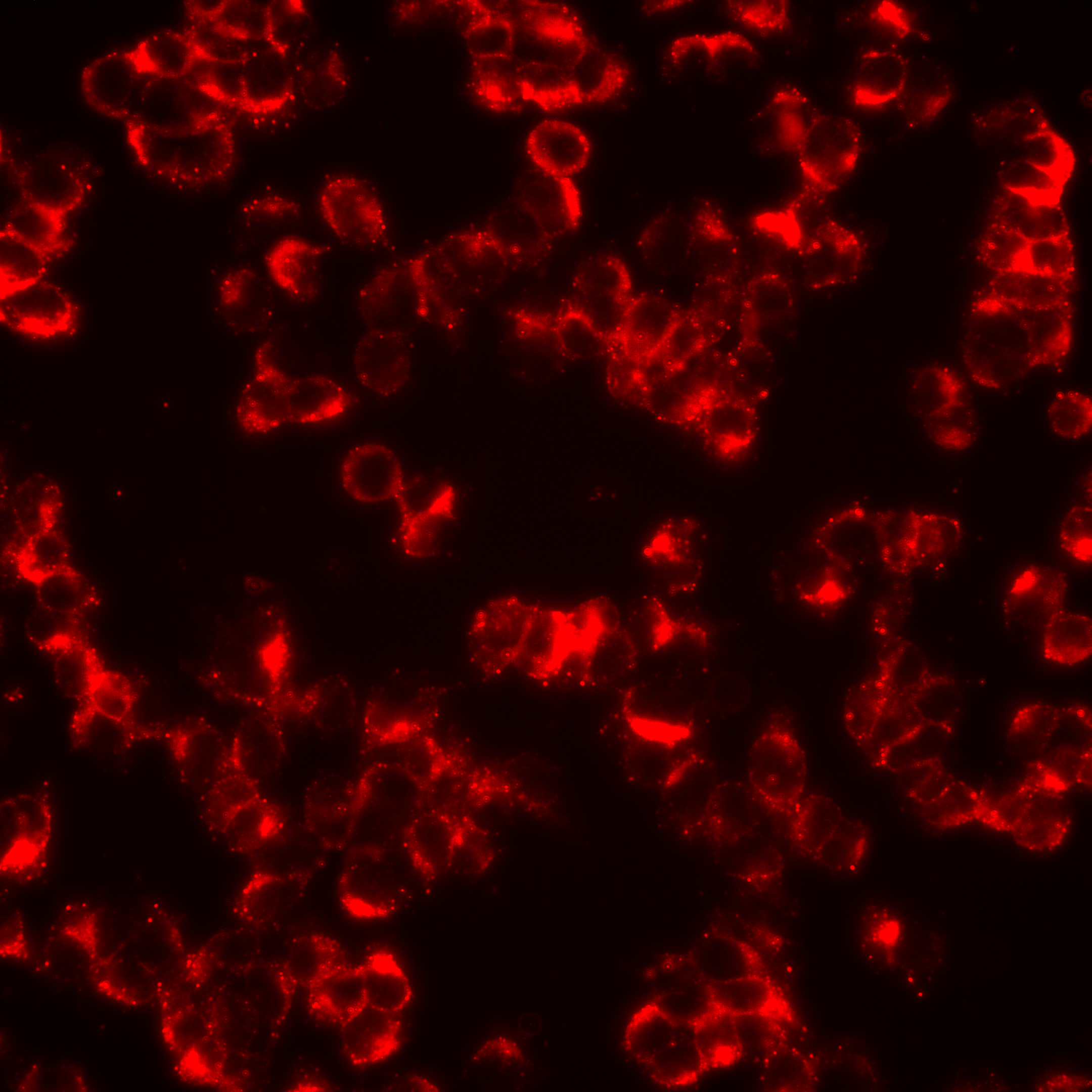

Supplement: Supplemental Information 4 [file peerj-04-1835-s004.tif]

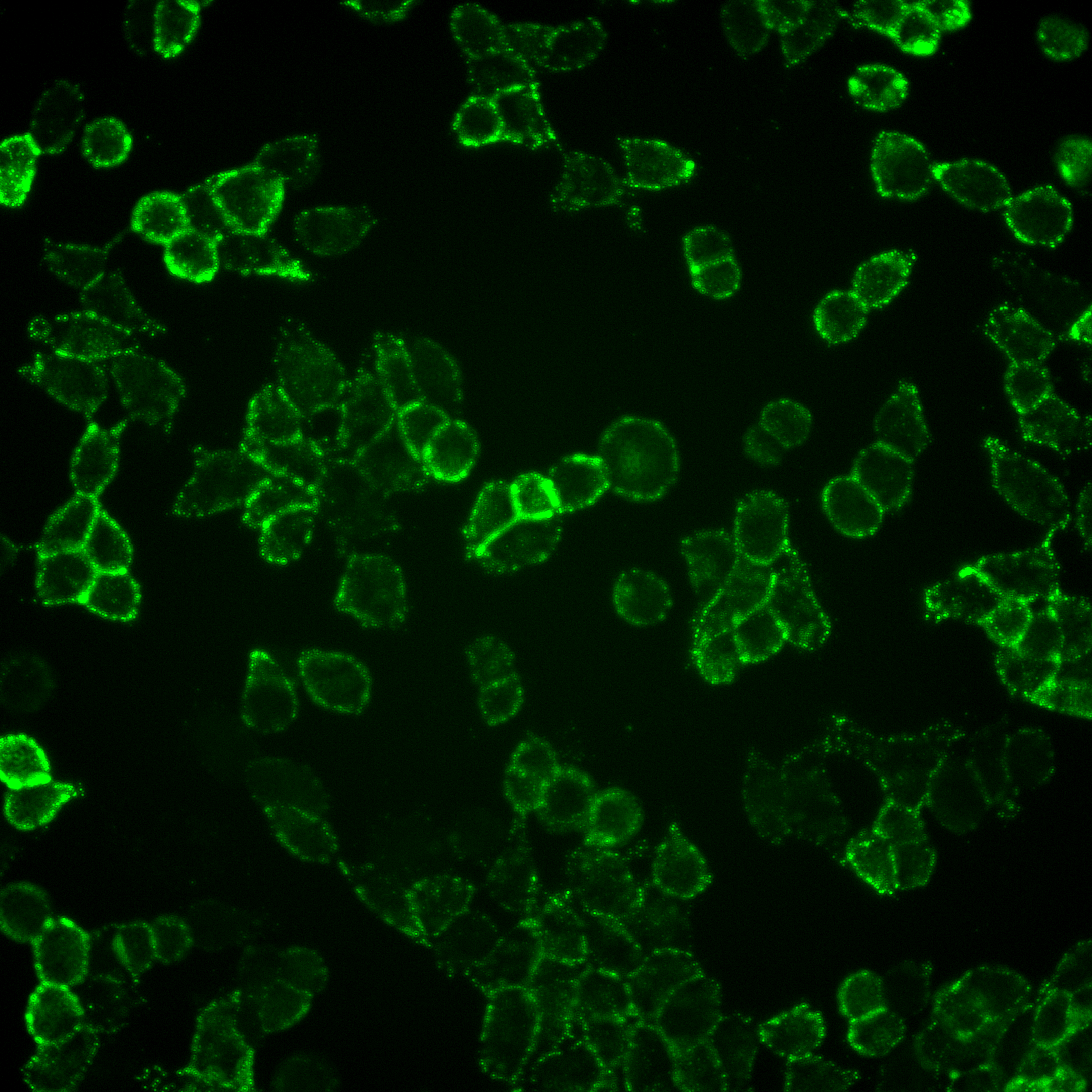

Supplement: Supplemental Information 5 [file peerj-04-1835-s005.tif]

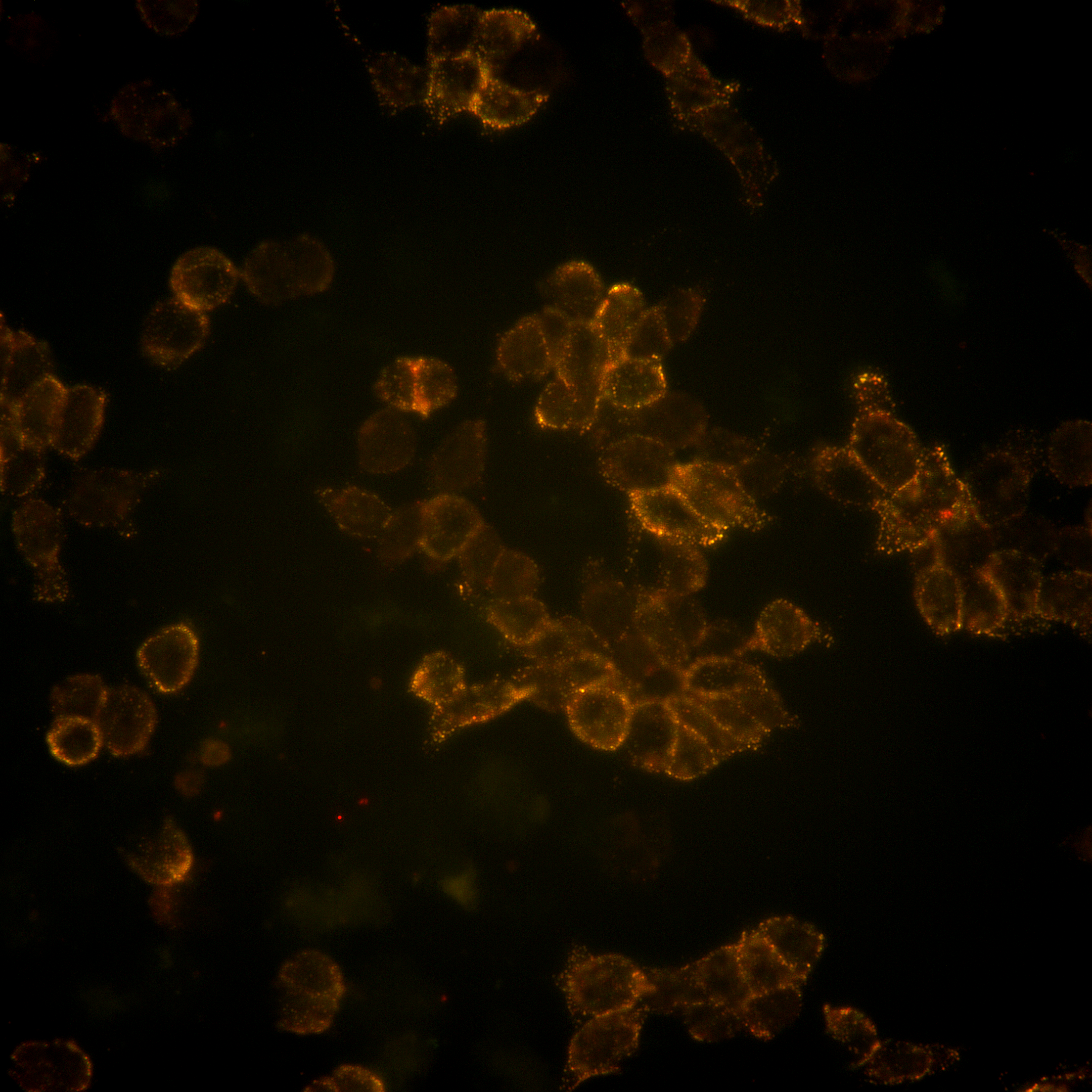

Supplement: Supplemental Information 6 [file peerj-04-1835-s006.tif]
